# Supplementary material for: The Mining of Genetic Loci and the Analysis of Candidate Genes to Identify the Physical and Chemical Markers of Anti-Senescence in Rice
Source: Plants (Basel). 2023 Nov 9;12(22):3812. doi: 10.3390/plants12223812 (PMC10674301; doi:10.3390/plants12223812)
Supplement: Supplementary file 1 [file plants-12-03812-s001.zip › plants-2657303-supplementary.pdf]

SUPPLEMENTARY MATERIALS FIGURE METHOD TABLE

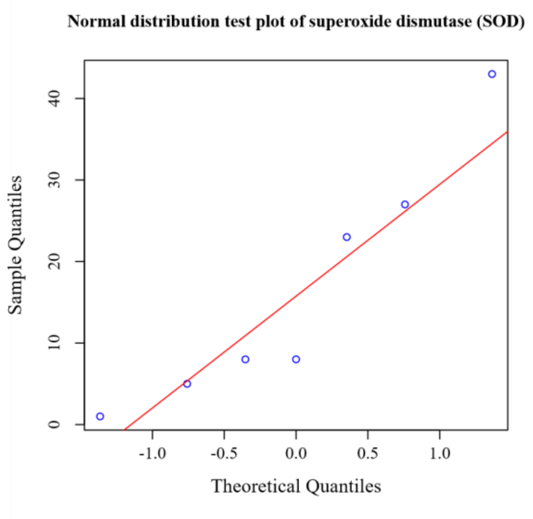

Figure S1. Normal distribution test plot of superoxide dismutase (SOD)

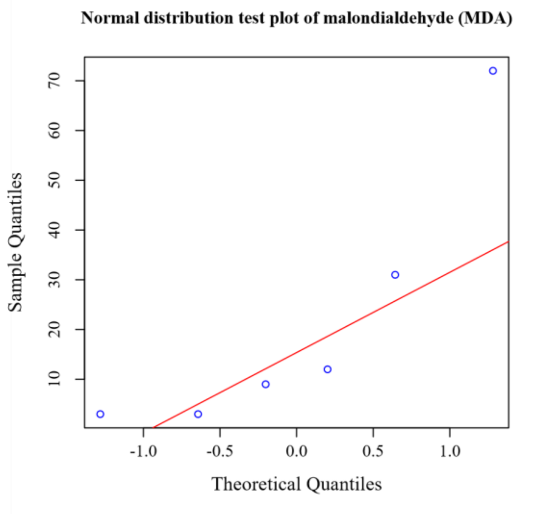

Figure S2. Normal distribution test plot of malondialdehyde (MDA)

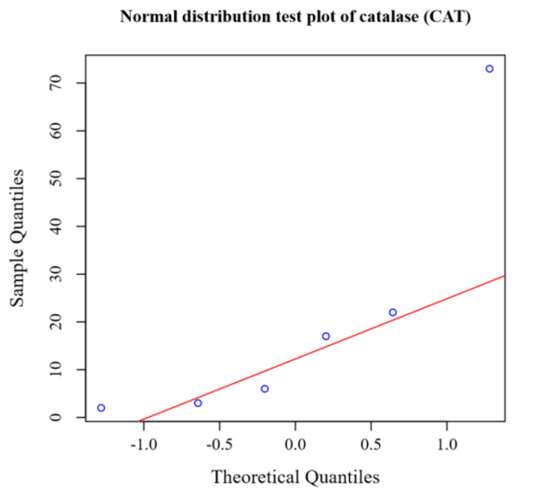

Figure S3. Normal distribution test plot of catalase (CAT)

Method S1. Methods for the determination of superoxide dismutase (SOD)

1. Reagent composition and preparation:

	Composition of reagents	A001-3-1: 48T	A001-3-2: 96T	Preservation
Reagent 1	Buffer solution	15 mL/bottle	30 mL/bottle	2~8°C for 6 months
Reagent 2	Substrate storage solution	0.07 mL/branch	0.15 mL/branch	2~8°C for 6 months
Preparation of substrate application solution: substrate storage solution : Buffer solution was mixed at a ratio of 1:200 to form substrate application solution, which was prepared immediately for use.				
Reagent 3	Enzyme storage solution	0.15 mL/branch	0.30 mL/branch	Below -20°C for 6 months
Reagent 4	Enzyme diluent	2 mL/bottle	4 mL/bottle	2~8°C for 6 months
Preparation of the enzyme working solution: enzyme storage solution : the enzyme dilution solution was mixed at a ratio of 1:10 to make the enzyme working solution, which was prepared immediately for use.				

2. Sample pretreatment: Take an appropriate amount of rice leaves with clean surface and no dirt in a mortar, add an appropriate amount of liquid nitrogen, quickly grind them into powder, and weigh the powder without leaves. According to the ratio of weight (g) : volume (mL)=1:9, 9 times the volume of phosphate buffer solution (0.1 mol/L pH=7~7.4) was added, vortexed and mixed for 3 minutes, then centrifuged at 4000 rpm 4°C for 10 minutes, and the supernatant (10% of the supernatant homogenate sample to be tested) was taken.

Note: The supernatant after centrifugation of plant tissue homogenate should be as clear and transparent as possible, and the centrifugation speed and time can be increased according to the actual situation.

3. Sample addition was determined according to the table below:

	Control hole	Control blank hole	Determination hole	Determination blank hole
Sample to be tested/μL	-	-	20	20
Distilled water/μL	20	20	-	-
Enzyme working solution/μL	20	20	20	-
Enzyme diluent/μL	-	20	-	20
Substrate application solution/μL	200	200	200	200
The mixture was mixed and incubated at 37°C for 20 min, followed by reading on a microplate reader at 450 nm.				

4. Formula of calculation:

1) SOD inhibition rate (%) = $\frac{(A_{control} - A_{controlblank}) - (A_{determination} - A_{determination\ blank})}{(A_{control} - A_{controlblank})} \times 100\%$

2) SOD activity (U/g tissue) = SOD inhibition rate ÷ 50% × $\frac{\text{system of reaction}}{\text{multiple of dilution}}\left(\frac{0.24\,mL}{0.02\,mL}\right)$ × dilutions before testing ÷ $\frac{\text{weight of tissue (g)}}{\text{the added homogenization buffer (mL)}}$

Method S2. Methods for the determination of malondialdehyde (MDA)

1. Reagent composition and preparation:

	Composition of reagents	Reagent specification	Preservation
Reagent 1	Clarifying agent	3 mL/bottle	Store at room temperature for one year
The reagent will solidify when it is cold during the day. Before each test, heat the water bath at 37°C to accelerate the dissolution until it is clear.			
Reagent 2	Buffer agent	45 mL/bottle	Refrigerate at 4 °C for one year
Reagent 3	Color development agent	15 mL/bottle	Refrigerate at 4 °C in the dark for one year
Working solution preparation: reagent 1: reagent 2: reagent 3 = 0.1:3:1 ratio for preparation, which was prepared immediately for use.			
Reagent 4	10 nmol/mL standard material	1 mL/bottle	Seal and refrigerate at 4 °C for one year
Reagent 5	Concentrate the extract 10 times	20 mL/bottle	Seal and refrigerate at 4 °C for one year
Reagent 5 preparation of the application extract: The ratio of 10 times concentrated extract: distilled water = 1:9 was prepared to form the application extract.			

2. Sample pretreatment: The plant tissue was weighed accurately, and 9 times the volume of reagent solution was added according to the weight/volume ratio of 1:9. After being cut into pieces, the homogenate was homogenized under an ice bath at 10000 rpm, each time lasting 10-15 seconds, intermittent 30 seconds, a total of 5 times. Then the homogenate was sucked into a centrifuge tube, centrifuged at 4000 rpm for 10 minutes, and the supernatant was taken for testing.

3. Sample addition was determined according to the table below:

	Blank tube	Standard tube	Determination tube
Absolute ethanol/μL	50	-	-
10 nmol/mL standard material/μL	-	50	-
Sample to be tested/μL	-	-	50
Working fluid/μL	1000	1000	1000
Cover the centrifuge tube (cover with a small hole with a needle), mix with vortex mixer, water bath above 95°C for 20 minutes, take it out and cool with running water, the wavelength is 530 nm, the microplate empty plate is read, 0.25 mL of each tube reaction liquid is accurately sucked into the 96-hole microplate, the microplate reader is used to measure the absorbance of each hole (the reading of the empty plate is subtracted from the calculation).			

4. Formula of calculation:

$$\text{Content of MDA (nmol/g)} = \frac{A_{determination} - A_{blank}}{A_{standard} - A_{blank}} \times C_{standard} \div \frac{W}{V_{extraction}}$$

Note: $C_{standard}$: concentration of standard, 10 nmol/mL

W: weight of plant tissue/g

$V_{extraction}$: the total amount of the extract/mL

Method S3. Methods for the determination of total protein (Cpr)

1. Reagent composition and preparation:

	Composition of reagents	Reagent specification	Preservation
Reagent 1	Coomassie brilliant blue stock solution	60 mL/bottle	Store at 4°C for six months
Preparation of Coomassie brilliant blue chromogenic solution: according to the ratio of Coomassie brilliant blue storage solution: distilled water =1:4 (5 times dilution), which was prepared immediately for use.			
Reagent 2	Protein standard solution	0.5 mL/branch	Store at 4°C for one month
If you want to prolong the storage time of reagent 2 (concentration see label), please divide the standard solution and freeze it at -20°C for 6 months.			

2. Sample pretreatment: The weight of the tissue to be tested was accurately weighed, and 9 times the volume of 0.9% saline was added according to the ratio of weight (g) : volume (mL) = 1:9, homogenized under an ice bath, centrifuged at 2500 rpm for 10 minutes, and the supernatant (10% homogenate) was taken to be measured.

3. Sample addition was determined according to the table below:

	Blank tube	Standard tube	Determination tube
Distilled water/mL	0.05	-	-
Protein standard solution/mL	-	0.05	-
Sample/mL	-	-	0.05
Coomassie brilliant blue chromogenic solution/mL	3.0	3.0	3.0
The mixture was mixed and allowed to stand for 10 minutes at 595 nm, 1 cm optical diameter, and distilled water was zeroed, and the OD value of each tube was measured.			

4. Formula of calculation:

$$\text{Protein of the sample to be tested (g/L)} = \frac{A_{determination} - A_{blank}}{A_{standard} - A_{blank}} \times C_{standard} \times N$$

Note: N represents the dilution of the sample before testing.

Method S4. Methods for the determination of catalase (CAT)

1. Reagent composition and preparation:

	Composition of reagents	Reagent specification	Preservation
Reagent 1	Liquid	100 mL/	Store at 4°C
Reagent 2	Liquid of substrate	10 mL/bottle	Store at 4°C
Reagent 3	Color developing powder	a bottle	Store at 4°C
Reagent 3 was dissolved by adding 100 mL of distilled water before use.			
Reagent 4	Liquid	10 mL/bottle	Store at 4°C
The reagent 4 will solidify when it is cold, and it can be used by heating it to transparency at 37°C before use.			

2. Sample pretreatment: The tissue was weighed accurately, and 9 times the volume of normal saline was added according to the ratio of weight (g) : volume (mL) = 1:9. Under the condition of

ice water bath, 10% tissue homogenate was prepared, centrifuged at 2500 rpm for 10 minutes, the supernatant was taken, and then diluted with normal saline to obtain the optimal sampling concentration.

3. Sample addition was determined according to the table below:

	Blank tube	Determination tube
Tissue homogenate/mL	-	0.05
Reagent 1/mL (preheat at 37°C)	1.0	1.0
Reagent 2/mL (preheat at 37°C)	0.1	0.1
The mixture was immediately mixed and timed, and the reaction was accurate for 1 min (60 seconds) at 37°C		
Reagent 3/mL	1.0	1.0
Reagent 4/mL	0.1	0.1
Tissue homogenate/mL	0.05	-
The mixture was mixed, the wavelength was 405 nm, the optical diameter was 0.5 cm, and the distilled water was zeroed. The absorbance value of each tube was measured and calculated $\Delta A_{Tissue\ homogenate} = A_{control} - A_{determination}$.		

4. Formula of calculation:

CAT activity in tissues (U/mgprot) = $\Delta A \times 271 \div V_{sample} \div T \div Cpr$

Note: 271 is the inverse of the slope, constant, and is used directly

V_{sample}: amount, 0.05 mL

Cpr: protein concentration in the homogenate, mgprot/mL (prot refers to protein)

Table S1. Anti-senescence candidate gene primers used for qRT-PCR

QTL	Gene ID	Forward primer (5’→3’)	Downstream primer (5’→3’)
	OsActin	TGGCATCTCAGCACATTCC	TGCACAATGGATGGGTCAGA
qCAT1	*LOC_Os01g61500*	AACTGGCGTAGAGCCTGAAA	ACAGCCTCACAAGCCTTCAT
	LOC_Os01g61810	ACAGCCTCACAAGCCTTCAT	ACTCCTCAAAGCCAAGCGTA
qMDA4	*LOC_Os04g40130*	AGGATGCTCTGAGGATTCGG	ACGCTCGTCTTCAGTGGTAA
qSOD8	*LOC_Os08g37760*	GTCATTGCCTCGCTGGTC	GCCGGATGTAGATCGAGAAG
	LOC_Os09g16920	TTCTACCCGGTCATGTGCTT	CTGAACATGCTGAATCCCGG
qMDA9.2	*LOC_Os09g16950*	CATGGTTGGTGTGATAGCCG	TGGTGTTGTACGACGAGTCA
	LOC_Os09g17010	GCCATCCTAGAGCTCCACAT	ATTCGCCGGTTTGATGTACC
qMDA11	*LOC_Os11g40690*	CACTGCTCGAAATGTGTCGT	AATGACGCCAAGTGACTTCC
qSOD12	*LOC_Os11g40750*	CCTGAGGATCCACTTTTCCA	TCGAAGCTGACACACTTTGG
	LOC_Os12g35330	TGGACCGTGTGATGAAGCTA	AGCTTCCCACCAATGAACAC
